# Sustainable Extractions for Maximizing Content of Antioxidant Phytochemicals from Black and Red Currants

**DOI:** 10.3390/foods11030325

**Published:** 2022-01-24

**Authors:** Anita Milić, Tatjana Daničić, Aleksandra Tepić Horecki, Zdravko Šumić, Nemanja Teslić, Danijela Bursać Kovačević, Predrag Putnik, Branimir Pavlić

**Affiliations:** 1 Faculty of Technology, University of Novi Sad, Bulevar Cara Lazara 1, 21000 Novi Sad, Serbia; anitavakula@uns.ac.rs (A.M.); tatjanadanicic@uns.ac.rs (T.D.); tepical@uns.ac.rs (A.T.H.); sumic@uns.ac.rs (Z.Š.); 2 Institute of Food Technology, University of Novi Sad, Bulevar Cara Lazara 1, 21000 Novi Sad, Serbia; nemanja.teslic@fins.uns.ac.rs; 3 Faculty of Food Technology and Biotechnology, University of Zagreb, Pierottijeva 6, 10000 Zagreb, Croatia; dbursac@pbf.hr; 4 Department of Food Technology, University North, Trg Dr. Žarka Dolinara 1, 48000 Koprivnica, Croatia

**Keywords:** black and red currant, solid–liquid extraction, ultrasound-assisted extraction, microwave-assisted extraction, pressurized-liquid extraction, phytochemicals

## Abstract

Sustainable extraction techniques (ultrasound-assisted extraction (UAE), microwave-assisted extraction (MAE), and pressurized-liquid extraction (PLE)) were applied and compared with conventional solvent extraction to evaluate their efficiency in maximizing the bioactive compound content and antioxidant activity of black and red currants. The influence of ethanol concentrations (30%, 50%, 70%) were studied in all extraction methods, while different temperatures (30, 50, 70 °C/80, 100, 120 °C) were evaluated in UAE and PLE, respectively. Generally, higher total phenolics were determined in black currant extracts (1.93–3.41 g GAE/100 g) than in red currant extracts (1.27–2.63 g GAE/100 g). The results showed that MAE was the most efficient for the extraction of bioactives from black currants, with 3.41 g GAE/100 g and 0.7934 g CE/100 g, while PLE provided the highest TP and TF for black currant samples (2.63 g GAE/100 g and 0.77 g CE/100 g). Extracts obtained by MAE (10 min, 600 W, 30% ethanol) and PLE (50% ethanol, 10 min, 120 °C) had the highest antioxidant activity, as determined by various in vitro assays (DPPH, FRAP, and ABTS). In conclusion, sustainable extraction techniques can be considered an efficient tool to maximize the content of bioactive antioxidants from black and red currants.

## 1. Introduction

Red (*Ribes rubrum* L.) and black (*Ribes nigrum* L.) currants are the two main berry species within the genus *Ribes*, which belongs to the widely distributed family *Grossulariaceae* [1]. The early 20th century was the time when mass cultivation of currants began in Europe. Nowadays, the global production of currants is still increasing [2], especially in areas with cooler climates suitable for commercial cultivation [3].

Currants are very important berries for the food industry, mainly because of their rich nutritional composition and attractive sensory properties [4]. Since the shelf life of these small and soft berries is usually short, most of the fruits are consumed in processed form, such as jams, jellies, juices [5], syrups, purees, and various ready-to-drink beverages [6]. In addition, currants could be an important ingredient in functional foods [7] and dietary supplements [8]. The quality of red and black currants is evaluated on the basis of their sensorial, nutritional, and processing properties as well as their potential biological characteristics [9]. Additionally, currants are recognized as a valuable source of bioactive compounds, especially polyphenolics (flavonoids, anthocyanins, flavonols), organic acids, and vitamins [4,10]. Among berries, currants stand out as fruits with by far the highest content of vitamin C [1], providing about five times higher content of this vitamin compared to citrus fruits [11]. These bioactive compounds are associated with significant antioxidant properties, being able to scavenge free radicals, in particular, breaking their chain reactions and thus preventing the occurrence of oxidative stress in the human body. Considering their enormous importance, the effects of processing and storage on the preservation of the aforementioned antioxidants with their health-promoting properties have been extensively studied [12].

Since extraction is the first step in the study of the biological compounds from a plant material, selecting the potentially most efficient extraction technique for a given raw material is always a challenging task [13]. Various classical extraction techniques applied to plant raw materials, so far, are usually based on solid–liquid extraction, using suitable organic solvents such as methanol and ethanol. The extraction power of the mentioned organic solvents and the possible application of heat, with or without mixing, were the main factors considered [14]. Due to their simplicity, relatively good efficiency, and wide application, conventional extraction techniques have been successfully used for the isolation of antioxidants (e.g., polyphenols, ascorbic acid) from berry extracts [15].

Recently, there has been a need to develop new environmentally friendly technologies in order to use them safely and reduce their impact on the environment [16]. These environmentally friendly and food-grade methods are relatively new alternative technologies that could maximize the extraction yield of biologically active compounds [17]. Here, innovative extraction methods should aim to improve the efficiency of the extraction with proportionally shorter processing time for isolation of bioactive compounds as well as lower consumption of water, energy, and organic solvents, which inevitably contribute to a much better environmental performance [18]. Different advanced extraction techniques, such as ultrasound-assisted extraction (UAE), microwave-assisted extraction (MAE), and pressurized-liquid extraction (PLE), have been shown to have great potential for application in the food industry, either alone or in combination, via the hurdle concept [19]. The application of these processing techniques has shown that they have great potential for replacing or modifying conventional extractions of naturally derived food ingredients [20].

Facilitated release of bioactive compounds during the application of UAE is enabled by the action of high-power ultrasonic waves and the occurrence of cavitation effects [21]. Increased solvent diffusion rates and disruption of the food matrix improve the solubility of the studied phytochemicals due to the influence of microwave power and significantly accelerate the extraction process. In addition to the ultrasonic/microwave power, the most important factors affecting ultrasound- and microwave-assisted extractions are the solvent-to-solid-matrix ratio, temperature, time, and the properties of the matrix and solvent [22]. In line with that, the aforementioned sustainable extractions were successfully used to improve the recovery of the total content of phytochemicals with high antioxidant activity from the blueberry samples studied [23,24]. In PLE, as an advanced extraction technique, the solvent is kept liquid throughout the extraction under high temperatures and pressures. This fast, efficient, selective, and sustainable method has been investigated for obtaining bioactive compounds from various fruit matrices [25,26,27,28]. Nevertheless, the conventional extractions are considered as the baseline method; hence, the efficacy and potential application of each newly developed technique are evaluated against them [14].

As far as we know, there are no reported data in the scientific literature on a comparative analysis of conventional (solvent extraction method) and sustainable extraction techniques (UAE, MAE, and PLE) applied with the aim of isolating the bioactive compounds from red and black currant fruits. Therefore, the main objective of this study is the extraction of bioactive molecules from dried red and black dried currants with the above techniques and their comparison in terms of maximizing the content of phytochemicals (total phenolic content, total flavonoid content, and total monomeric anthocyanin content) as well as total yield and antioxidant activity.

## 2. Materials and Methods

### 2.1. Sample

Samples of black and red currants were purchased at a local market (Novi Sad, Serbia). All samples were stored in a freezer (−20 °C) until vacuum drying. Vacuum drying was performed in a prototype vacuum dryer with a vacuum pump, installed at the Faculty of Technology Novi Sad, Serbia. Details of the dryer used are described in detail in the article by Šumić et al. [29]. The drying conditions were chosen following previous studies on red currant drying, as presented in Vakula et al. [3]. There, it was found that the sample dried at 60 °C, 20 mbar, and in 16 h had the best quality indicators and the best economy of process. Therefore, these conditions were chosen for drying black and red currants in this study. The moisture content of the vacuum-dried sample of red currants was 9.23%, and the moisture content of the vacuum-dried sample of black currants was 11.48%.

### 2.2. Reagents

The following reagents were purchased from Sigma-Aldrich Chem (Steinheim, Germany): Folin–Ciocalteu reagent; (±)-catechin; gallic acid; 2,2-diphenyl-1-picrylhydrazyl (DPPH); 2,4,6-tris (2-pyridil)-s triazine (TPZT); iron (III)-chloride and iron (II)-sulfatheptahydrate and potassium persulfate. Trolox (6-hydroxy-2,5,7,8-tetramethylchroman-2-carboxylic acid) was obtained from Sigma-Aldrich (Milan, Italy). Sodium acetate and hydrochloric acid were purchased from Merck (Darmstadt, Germany). ABTS (2,2′-azino-bis-(-3-ethylbenzothiazoline-6-sulfonic acid) diammonium salt) was purchased from J&K Scientific GmbH (Pforzheim, Germany). All other chemicals and solvents were of analytical reagent grade.

### 2.3. Extraction Techniques

#### 2.3.1. Solid–Liquid Extraction

Solid–liquid (S/L) extraction was performed conventionally. In each experimental run, samples with 5.0 g of dried black and red currants were extracted in 50 mL of hydroalcoholic solvent using different concentrations of ethanol (30%, 50%, and 70%, *v*/*v*) as the extraction solvent. The extractions were carried out at room temperature and lasted for 24 h at a stirring speed of 150 rpm. After processing, the extracts were immediately filtered through a vacuum filter (V-700, BÜCHI Labortechnik AG, Flawil, Germany) and then collected in glass vials and stored at 4 °C until analysis.

#### 2.3.2. Pressurized-Liquid Extraction (PLE)

Pressurized-liquid extraction (PLE) was performed using an accelerated solvent extractor (Dionex™ ASE™ 350, Sunnyvale, CA, USA), following the method previously described by Mrkonjić et al. [30]. In each experimental run, 5 g of the dried black and red currants and 1 g of diatomaceous earth as desiccant were mixed and added to a 22 mL stainless steel extraction cell. Experiments were performed at a fixed pressure (10.34 MPa) and a fixed purge time with N_2_ (90 s). The ethanol concentration (30%, 50%, and 70%, *v*/*v*) and temperature (80, 100, and 120 °C) were varied in the screening experiments. The static extraction time was 10 min for all experiments, while the number of cycles was 2 for all experiments with 100% rinse. The extracts were diluted with a solvent to adjust the solid-to-liquid ratio to 1:20 (*w*/*v*). The obtained samples were then collected into plastic vials and stored at 4 °C prior to analysis.

#### 2.3.3. Microwave-Assisted Extraction

Microwave-assisted extraction (MAE) was performed in mono-mode at a fixed frequency. The homemade MAE setup consisted of a microwave oven (MM817ASM, Bosch, Munich, Germany), a glass apparatus with a round bottom flask of 500 mL, and a reflux condenser, according to the method described by Pavlić et al. [31]. Briefly, for each experiment, 5 g of the dried black and red currants in a volume of 50 mL of solvent was used. The independent MAE variables were ethanol concentration (30%, 50%, and 70%), the extraction time for each experiment was 10 min, and the irradiation power (600 W) was kept constant. In all experiments, the flasks were positioned in the same position of the microwave extractor, and no additional stirring was applied, while the temperature in the flask was at the boiling point, depending on the applied mixture of water and ethanol. After extraction, the crude extracts were immediately filtered through filter paper (4–12 μm pore size, Schleicher & Schuell, Darmstadt, Germany) under vacuum (V-700, Büchi, Switzerland). The extracts were then collected in glass flasks and stored at 4 °C until analysis.

#### 2.3.4. Ultrasound-Assisted Extraction

For ultrasound-assisted extraction (UAE), an ultrasound water bath device (EUP540A, Euinstruments, Paris, France) operating at a fixed frequency (40 kHz) was used. Experiments were performed according to a modified UAE procedure applied for the recovery of polyphenols from cherries [32]. In each experiment, 5.0 g of dried black and red currants was mixed with 50 mL of extraction solvent (30%, 50%, and 70%) in 300 mL glass flasks. In all experiments, the flasks were positioned at the same distance from the transducer, and no additional stirrer was used either. Different temperatures were used (30, 50, and 70 °C), while the other parameters were time (30 min) and ultrasonic power (60 W/L). After extraction, the extracts were immediately filtered through a vacuum filter. The extracts were then filled into glass vials and stored at 4 °C until analysis.

### 2.4. Experimental Design

Dried samples of black and red currants were extracted using different drying techniques: S/L extraction, UAE, MAE, and PLE and different extraction conditions (ethanol concentration and temperature). A total of *n* = 32 extracts were made, and the experimental design is shown in Table 1.

### 2.5. Extraction Yield

The content of yields in the obtained extracts of red and black currants was obtained by the procedure of vacuum vaporization using 10 mL of the crude extract. After vaporization, drying was carried out in an oven (Sutjeska, Sutjeska, Serbia) at 105 °C until a constant mass was obtained. Results are expressed as mass of total extractable solids per 100 g of dry plant material (%; *w/w*).

### 2.6. Analyses of Bioactive Compounds

Total phenolic content was determined by Folin–Ciocalteu colorimetric assay [33], where gallic acid was used as standard with measured absorbance at 750 nm (6300 Spectrophotometer, Jenway, Stone, UK). Total flavonoid content was determined using the aluminum chloride colorimetric test [34], where catechin was used for the preparation of the standard diagram and absorbance was measured at 510 nm. Total monomeric anthocyanins content was determined according to the pH differential method described by Fuleki and Francis [35]. Two buffer systems (potassium chloride buffer, pH 1.0 (0.025 M), and sodium acetate buffer, pH 4.5 (0.4 M)) were used. Total monomeric anthocyanins were expressed as mg cyanidin-3-glucoside/100 g of dry fruit weight (mg CGE/100 g) and calculated as follows:

(1) $$\frac{A\;\times \mathrm{MW}\;\times \mathrm{DF}\;\times 10^{3}}{\varepsilon \times l}$$

where A = (A_520nm_ − A_700nm_) _pH = 1.0_ − (A_520nm_ − A_700nm_) _pH = 4.5_; MW = molecular weight (for cynidin-3-glucoside C_21_H_21_ClO_10_ = 484.8 g mol^−1^); DF = dilution factor; 10^3^ = factor for conversion g to mg; ε = molar absorption extinction coefficient (for pelargonidine-3-glucoside 22400 L mol^−1^ cm^−1^); l = cuvette thickness (1 cm).

Each measurement was carried out three times, and results are presented as mean values.

### 2.7. Analyses of Antioxidant Activity

The ability of the extracts to scavenge free radicals of 2,2-diphenyl-1-picrylhydrazyl (DPPH·) was measured using a slightly modified method originally presented by Brand-Williams et al. [36]. The reducing ability of the extracts towards Fe^3+^ was measured using the slightly modified method by Benzie and Strain [37]. The ability of the extracts to scavenge ABTS free radicals was measured using a modified method by Re et al. [38]. Each measurement was carried out three times, and the results are presented as mean values. A detailed description of all methods is described in the work of Milić et al. [32].

### 2.8. Statistical Analysis

All experiments were performed in triplicates. Results were expressed as mean ± standard deviation (SD). All data were analyzed using analysis of variance (ANOVA) with Tukey’s multiple comparison test at *p* < 0.05. Statistica 10.0 (StatSoft, Inc., Tulsa, OK, USA) was used for ANOVA.

## 3. Results and Discussion

### 3.1. Extraction Yield

**Figure 1:**
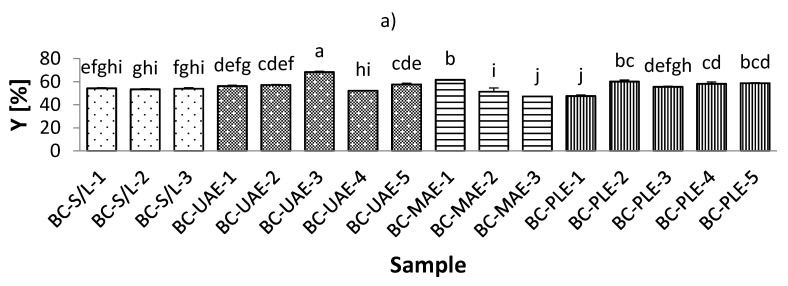

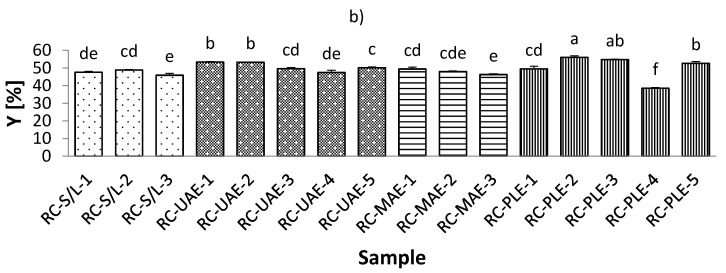
Total extraction yield (Y) of black (**a**) and red (**b**) currant extracts obtained by conventional and sustainable extraction techniques. RC—red currant extract; S/L—conventional solvent extraction, UAE—ultrasound-assisted extraction; MAE—microwave-assisted extraction; PLE—pressurized-liquid extraction. Results are expressed as mean ± standard deviation (SD). Tukey’s multiple comparison test was performed at *p* < 0.05, and different letters represent statistically significant differences among samples.

The total extraction yield (Y) of the black currant extracts varied from 47.19% to 68.43%, which was observed for extracts obtained by both MAE and UAE at 70% ethanol (Figure 1, Table S1; Supplementary Materials). On the other hand, the Y of red currant was lower and ranged from 38.49–56.04% in the extracts obtained by PLE (50% ethanol/80 °C; and 50% ethanol/100 °C, respectively). It was also found that the Y in the black currant extracts was generally higher than in the red currant extracts, indicating a higher content of bioactive compounds in the black currant. Regarding the influence of ethanol in black currant extracts, it was found that for S/L and MAE, the highest Y was obtained at the lowest applied ethanol concentrations (30% ethanol), while only for MAE, the lowest Y was obtained at the highest applied ethanol concentration (70% ethanol). This means that for MAE, the ethanol concentration is inversely related to the yield in the extracts, which is in agreement with previously reported findings [39]. For UAE and PLE black currant extracts, the lowest Y was found at the lowest ethanol concentration (30% ethanol), while UAE extracts were also found to have the highest Y obtained at the highest ethanol concentration applied (70% ethanol). This means that for PLE, yield and ethanol were proportionally related. Similarly, for red currant extracts with UAE and MAE, the highest Y was at the lowest ethanol concentration (30% ethanol).

The concentration of ethanol is one of the most important factors affecting yield in UAE [40]. An increase in ethanol concentration then negatively affects the yield. This variable trend could be explained by the increase in the solubility and diffusivity of the bioactive compound due to the decrease in the dielectric constant of the solvent with increasing ethanol concentration [40]. The main conclusion regarding the influence of the studied temperature on the Y of the extracts, for both black and red currant extracts, was that the lower Y was at lower temperatures, which is in agreement with previous results [41].

### 3.2. Total Phenolic Content

The antioxidant activity of phenolic compounds has been known for decades, and in recent years, the bioactivity of these compounds has become increasingly known. These compounds are widely used in plant foods and have been associated with the sensory and nutritional properties of processed plant foods [42]. According to this importance of phenolic compounds, they have been investigated by many authors in different foods and also in the black and red currants [43,44,45]. The lowest total phenolic content (TPC) in black currant extracts was found with S/L and 70% ethanol, while the highest TPC was obtained by MAE and 30% ethanol (Table S2; Supplementary Materials). These results regarding the lowest TPC in the S/L extracts were expected, considering the advantages (specificities) of ultrasound and microwave power and higher temperatures of PLE. On the other hand, both the lowest (80 °C) and the highest (120 °C) TPCs were in red currant extracts obtained by PLE. However, it should be noted that extracts obtained with S/L at 70% ethanol were similar to the lowest TPC of all red currant extracts as they were for the black currant extracts.

**Figure 2:**
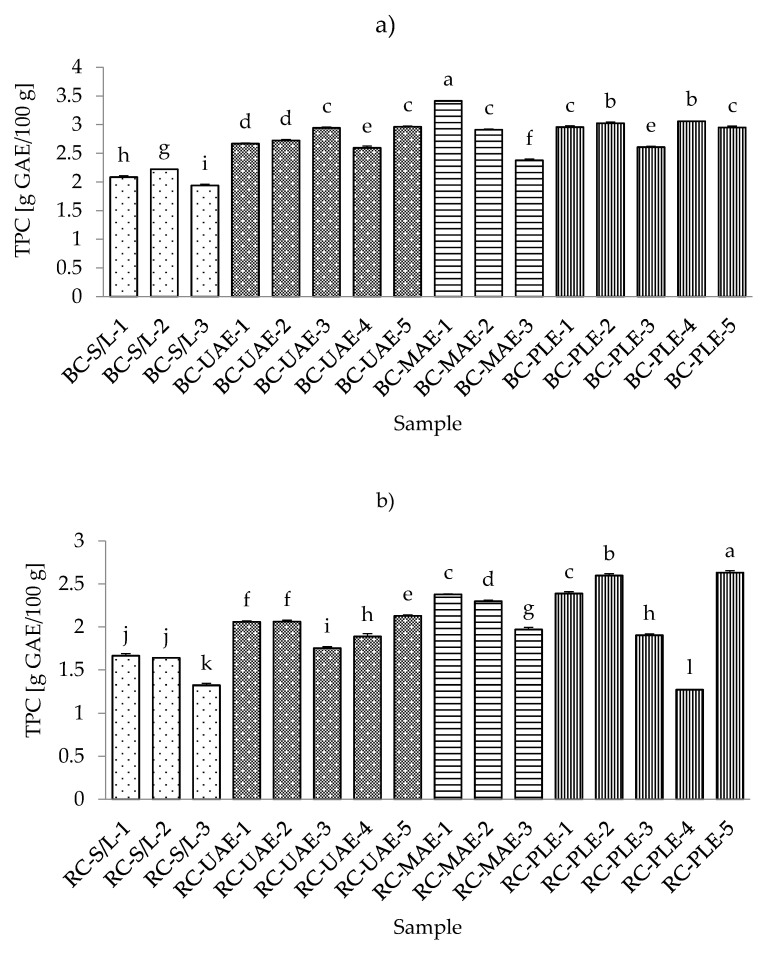
Total phenolic content (TPC) of black (**a**) and red (**b**) currant extracts obtained by conventional and sustainable extraction techniques. RC—red currant extract; S/L—conventional solvent extraction, UAE—ultrasound-assisted extraction; MAE—microwave-assisted extraction; PLE—pressurized-liquid extraction. Results are expressed as mean ± standard deviation (SD). Tukey’s multiple comparison test was performed at *p* < 0.05, and different letters represent statistically significant differences among samples.

In general, it could be seen from Figure 2a,b that both the red and black currant extracts obtained by S/L had lower TPCs, while the other three extractions had higher levels of TPC. In the study by Laczkó-Zöld et al. [10], even lower TPCs were found in black and red currant extracts obtained by S/L under different extraction conditions (ranging from 0.133–0.225 g/100 g FW and from 0.073–0.192 g/100 g FW). Lapornik et al. [46] presented the results of a comparison of extracts prepared from plant by-products using different solvents and extraction times, and it was found that the total content of polyphenols ranged from 2.43–9.70 g/L in black current and from 0.40–1.17 g/L in red currant by S/L extraction.

As mentioned earlier for different conditions of S/L extraction, it was found that for both red and black currants, the lowest TPC was obtained under the conditions with the highest ethanol concentration (70%), which was also the case for the techniques MAE and PLE for both black and red currants and also for UAE for red currant extracts. As the concentration of water in ethanol decreases (30% vs. 50%), the TPC in the extract increases. Similarly, optimization of phenolic extraction from aromatic and fruit-bearing tree leaves using hydroethanolic mixtures confirmed that the ethanol content of the extraction mixture affected the phenol recovery yield, while the highest TPC was not found in extracts with the highest ethanol concentrations [47]. This could be due to the formation of some phenolic compounds in the extract that are more soluble in water. These phenolic compounds may possess more phenol groups or have higher molecular weight than the phenols solved in ethanol [48]. Based on the results of TPC, the best extraction solvent was 50% ethanol.

When considering different extraction temperatures with the same values of other extraction conditions, it was found that for both red and black currant extracts obtained with UAE, the lower temperature (30 °C) yielded lower TPC and the higher temperatures (70 °C) yielded higher TPC for the same extraction conditions (e.g., m = 5 g sample, V = 50 mL solvent, t = 30 min, 50% ethanol, P = 60 W/L). The same was true for the red currant PLE, where a higher TPC was observed at a higher temperature (120 °C) and the lowest TPC was observed at a lower temperature (80 °C) under the same extraction conditions (m = 5 g sample, 2 cycles, 100% rinse, 50% ethanol, t (static extraction time) = 10 min). It is commonly believed that increasing the extraction temperature improves diffusivity, softens the plant tissue, and promotes elution of the bound phenols in the hydroethanolic mixture [47]. From the results for black currant PLE extracts and the influence of different temperatures under the same extraction conditions, it was found that the lower temperature (80 °C) did not significantly affect the TPC, while the higher temperature (120 °C) caused a lower TPC. However, higher temperatures increase the chance of the oxidation of phenolics, which may decrease their yield in the extracts [49].

### 3.3. Total Flavonoid Content

The range of total flavonoid content (TFC) in all black currant samples was from 0.4717 g CE/100 g for S/L with 70% ethanol up to 0.8321 g CE/100 g for UAE at 50 °C and 70% ethanol (Table S3; Supplementary Materials). The lowest content of flavonoids in all black currant extract samples is in agreement with the results obtained for total phenolic content. This could be expected since the flavonoids represent a subgroup of polyphenols [25] and extraction techniques, as well as the applied process parameters, occasionally affect the extraction of TP and TF similarly.

**Figure 3:**
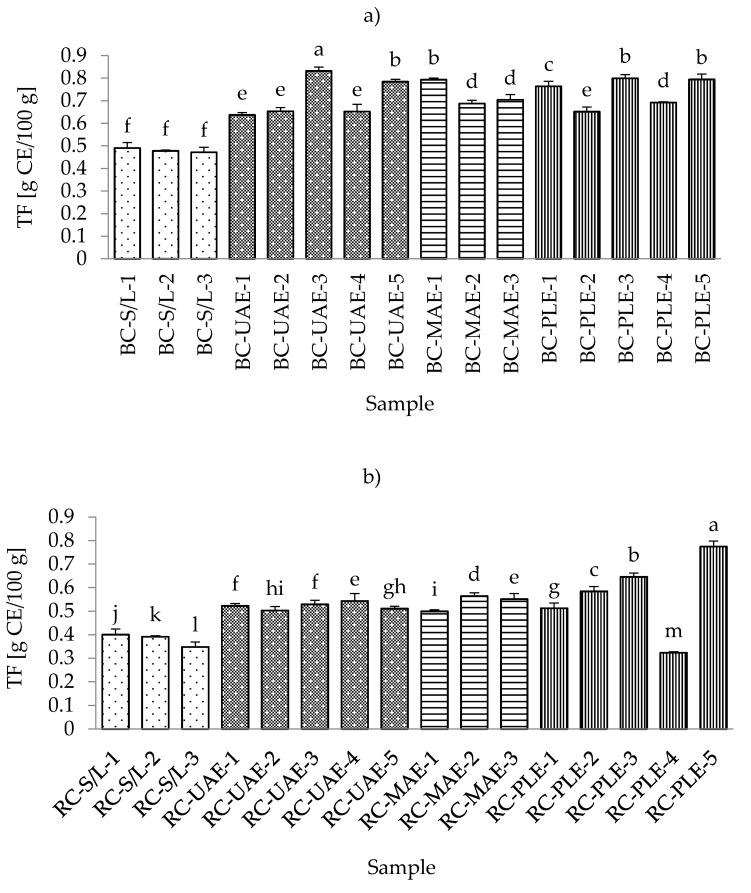
Total flavonoid content (TFC) of black (**a**) and red (**b**) currant extracts obtained by conventional and sustainable extraction techniques. RC—red currant extract; S/L—conventional solvent extraction, UAE—ultrasound-assisted extraction; MAE—microwave-assisted extraction; PLE—pressurized-liquid extraction. Results were expressed as mean ± standard deviation (SD). Tukey’s multiple comparison test was performed at *p* < 0.05, and different letters represent statistically significant differences among samples.

In the extract obtained by MAE (30% ethanol), where the highest TPC was obtained, a higher TFC was also detected. Furthermore, equal TFCs were detected in extracts obtained by MAE (50% ethanol), PLE (100 °C and 50% ethanol), and PLE (120 °C and 50% ethanol). In the case of red currant, the conclusions were the same as before with TPC since both the lowest and the highest content of TFC in red currant extracts were also obtained by PLE at 80 and 120 °C, respectively. Additionally, it was also found that the lowest TFCs were obtained in S/L extracts, as was the case for TPC and TFC in black and red currant extracts. Accordingly, the general conclusion could be the same as in the case of TPC, that S/L extraction resulted in significantly lower TFC compared to the other three investigated extraction techniques studied (Figure 3a,b).

In a study by Laczkó-Zöld et al. [10], lower TFC values were found in extracts of black and red currants obtained with S/L extraction under different extraction conditions (0.021–0.126 g/100 g FW; and 0.037–0.040 g/100 g FW, respectively). Regarding the influence of ethanol in the extraction process, it was noticed that both black and red currant extracts by S/L and MAE had the highest TFC value at the lowest ethanol concentration (30%), while UAE and PLE extracts had the lowest TFC value at the lowest ethanol concentration (30%) and the highest TFC value at the highest ethanol concentration (70%).

For UAE and PLE black currant extracts and for PLE red currant extracts, the higher applied temperatures (70 and 120 °C, respectively) yielded higher TFCs. For the lower applied temperatures in UAE and PLE (30 and 80 °C, respectively), for black currant extracts, it was noticed that the lower temperature even yielded a higher TFC that was not significantly different from UAE extracts. However, it significantly differed from the PLE, while in the case of red currants, lower temperature gave higher TFC for UAE; however, in the case of PLE, this influence was proportional, i.e., lower temperatures yielded lower TFCs.

### 3.4. Total Monomeric Anthocyanin Content

**Figure 4:**
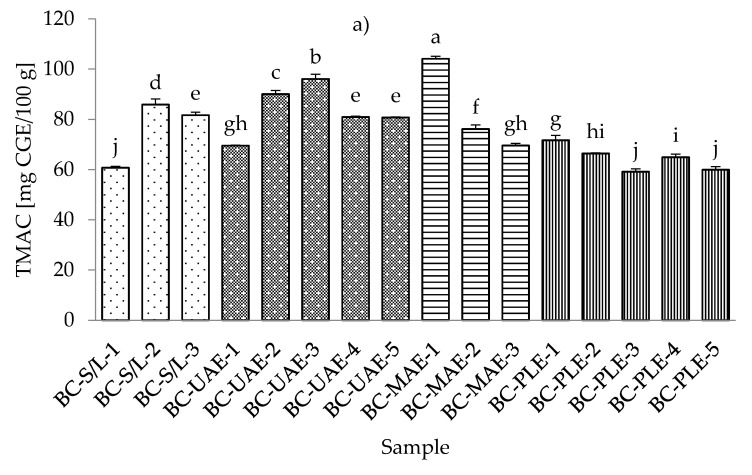

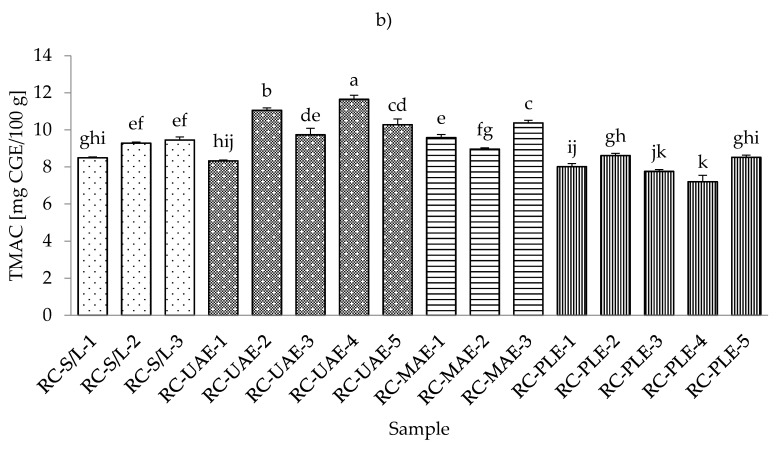
Total monomeric anthocyanin content (TMAC) of black (**a**) and red (**b**) currant extracts obtained by conventional and sustainable extraction techniques. RC—red currant extract; S/L—conventional solvent extraction, UAE—ultrasound-assisted extraction; MAE—microwave-assisted extraction; PLE—pressurized-liquid extraction. Results are expressed as mean ± standard deviation (SD). Tukey’s multiple comparison test was performed at *p* < 0.05, and different letters represent statistically significant differences among samples.

The lowest total monomeric anthocyanin contents (TMACs) from all the black currant extracts were determined with PLE (30% ethanol), while the highest TMAC was detected with MAE and even at the lowest applied ethanol concentration (30%) (Table S4; Supplementary Materials). It should be noted that the black currant extracts obtained by S/L (30% ethanol) also showed a very low TMAC content, which was in agreement with the results obtained for the total phenolic and flavonoid content. As for the red currant extracts, the lowest TMAC was obtained in the red currant PLE (50% ethanol, 80 °C), which was in agreement with the results for black currant, where the lowest TMAC was also obtained in PLE (Figure 4a,b).

In the study of Laczkó-Zöld et al. [10], higher TMAC values were found in the extracts of black and red currant with S/L extraction under different extraction conditions (from 187.66–327.34 mg CGE /100 g FW and from 17.66–21.26 mg CGE /100 g FW). In the work of Lapornik et al. [46], it was concluded that the anthocyanin content in black currant S/L extracts ranged from 1.79 to 6.81 g/L and in red currant extracts from 0.086 to 0.34 g/L. These were higher values compared to the TMAC obtained in the current study. However, the highest TMAC in red currant was obtained with UAE and 50% ethanol at 30 °C.

Regarding the influence of different ethanol concentrations, it was found that lower concentrations gave lower TMACs in black currant extracts in the case of S/L and UAE. In the case of UAE extracts, it was also found that higher TMACs were obtained at higher ethanol concentrations. In the case of MAE and PLE extracts of black currant, lower ethanol concentrations gave higher TMACs and vice versa; higher ethanol concentrations generated lower TMACs. For red currant extracts, lower ethanol concentrations generated lower TMACs in S/L red currant and UAE, while higher ethanol concentrations caused higher TMACs in red currant for S/L extracts.

For MAE and PLE extracts of red currant, ethanol concentration produced higher TMACs, while lower ethanol concentration gave higher TMACs. The effect of lower and higher applied temperatures was also different both for black and red currant extracts. For example, for both black and red currant extracts, a lower TMAC was observed in the extract obtained at a higher temperature, while in the case of PLE, lower temperatures produced lower TMACs of black and red currant extracts.

### 3.5. Antioxidant Activity

To get a complete overview of the antioxidant activity of all obtained extracts, three different antioxidant assays were performed, namely, DPPH, FRAP, and ABTS. The results presented in Table 2 and Table 3 suggest that for both black and red currants, a minimal antioxidant activity was detected in the S/L extracts with the maximum ethanol concentration of 70% (by DPPH and ABTS). Furthermore, maximum activity was detected for both black and red currants in extracts by PLE with the minimal ethanol concentration of 30% (by FRAP) and in extracts obtained by MAE, also with the same minimum ethanol concentration (by ABTS).

The antioxidant activity determined by the DPPH assay in the black currant extracts ranged from 1.77–3.67 IC_50_ mg/mL and in the red currant extracts from 5.72–34.26 IC_50_ mg/mL, although other extraction solvents were used during S/L [10]. Similar to the DPPH, the authors also confirmed that higher values were found in extracts with higher water content in the extraction solvent with ABTS [10]. The results of this study showed that a similar trend was observed, but only up to an ethanol concentration of 50%. For extracts with 70% ethanol, all values of antioxidant capacity of S/L extracts decreased.

In the work of Lapornik et al. [46], it was concluded that antioxidant activity ranged from 15.1% to 49.2% in black currant extracts and from 1.0% to 7.1% in red currant extracts obtained by S/L extraction at different conditions. In a study about different drying techniques of black and red currants, it was found in the S/L extraction that the highest ethanol concentration (70%) had the lowest antioxidant activity for all the techniques investigated and for all three antioxidant assays. The lowest antioxidant activity at the highest ethanol concentration (70%) and the highest antioxidant activity at the lowest ethanol concentration (30%) was detected for UAE, MAE, and PLE black currant extracts (by DPPH); similar findings were detected for MAE and PLE black currant extracts (by FRAP), for MAE black currant extracts, for S/L red currant extracts (by DPPH), for MAE and PLE red currant extracts (by FRAP), and for MAE red currant extracts (by ABTS).

Considering the extraction temperature and antioxidant activity, here, UAE and PLE extracts of black and red currants had lower antioxidant activity at lower temperatures under the same extraction conditions (by DPPH), while higher antioxidant activity was observed at higher temperatures. The same results were observed for the antioxidant activity of red currant (by FRAP and ABTS) and black currant (by ABTS), as higher temperatures generally caused higher antioxidant activity in these samples. It could be concluded that sustainable extraction techniques generally provided the black and red currant extracts with more potent antioxidant activity compared to conventional solid–liquid extraction. More specifically, BC-UAE-5 and RC-UAE-5 samples were the best set of UAE parameters (50% ethanol and 70°C). In the case of MAE and PLE, the results suggested that extractions should be performed with 30% ethanol as the extraction solvent in order to achieve the highest antioxidant activity since the most potent extracts obtained by these techniques were BC-MAE-1, RC-MAE-1, BC-PLE-1, RC-PLE-1, BC-PLE-5, and RC-PLE-5 (Table 2 and Table 3).

## 4. Conclusions

The results of this study confirm that UAE, MAE, and PLE represent sustainable extraction technologies for the production of high-value extracts from red and black currants using aqueous ethanol as an extraction solvent. Generally, the results imply that the maximum contents of all investigated parameters in both black and red currants are with UAE, where black currant extracts had the highest total flavonoid content, while red currant extracts had the highest monomeric anthocyanin content. In black currant extracts, MAE resulted in the maximum contents of total phenolics and flavonoids and the highest extraction yield and antioxidant activity (by ABTS). For the red currant extracts, PLE gave the maximum contents of total phenolics and flavonoids, extraction yield, and antioxidant activity (by FRAP). S/L extraction underperformed all other alternatives as none of the analyses managed to confirm the maximum contents for this extraction. Despite this, the results showed that for both dried black and red currants, all of the studied techniques resulted in high-quality extracts with respect to all studied parameters. The most suitable extraction technique for the blackcurrant was MAE, and for the red currant, it was PLE, in terms of maximizing both the studied contents of bioactive compounds and antioxidant activity.

## Figures and Tables

**Table 1:** The experimental design for S/L, UAE, MAE, and PLE of dried black and red currant samples.

Sample		Extraction Technique	Extraction Parameters	Factor
Black Currant	Red Currant			
BC-S/L-1	RC-S/L-1	S/L	1:10 (m:v) solid-to-liquid ratio, 24 h, room temperature, 150 rpm (shaker)	30% ethanol
BC-S/L-2	RC-S/L-2			50% ethanol
BC-S/L-3	RC-S/L-3			70% ethanol
BC-UAE-1	RC-UAE-1	UAE	1:10 (m:v) solid-to-liquid ratio, 30 min, 50 °C, 60 W/L (ultrasonic power), 40 kHz frequency	30% ethanol
BC-UAE-2	RC-UAE-2			50% ethanol
BC-UAE-3	RC-UAE-3			70% ethanol
BC-UAE-4	RC-UAE-4		1:10 (m:v) solid-to-liquid ratio, 30 min, 50% ethanol, 60 W/L (ultrasonic power)	30 °C
BC-UAE-5	RC-UAE-5			70 °C
BC-MAE-1	RC-MAE-1	MAE	1:10 (m:v) solid-to-liquid ratio, 10 min, 600 W (microwave power)	30% ethanol
BC-MAE-2	RC-MAE-2			50% ethanol
BC-MAE-3	RC-MAE-3			70% ethanol
BC-PLE-1	RC-PLE-1	PLE	5 g sample, 1:20 (m:v) solid-to-liquid ratio, 2 cycles, 100% rinse, 100°C, 10 min dynamic extraction time	30% ethanol
BC-PLE-2 BC-PLE-3	RC-PLE-2 RC-PLE-3			50% ethanol 70% ethanol
BC-PLE-4	RC-PLE-4		5 g sample, 1:20 (m:v) solid-to-liquid ratio, 2 cycles, 100% rinse, 50% ethanol, 10 min dynamic extraction time	80 °C
BC-PLE-5	RC-PLE-5			120 °C

**Table 2:** Antioxidant activity obtained by DPPH, FRAP, and ABTS assays of black currant extracts obtained by conventional and sustainable extraction techniques.

Sample	DPPH [µM TE/g]	FRAP [µM Fe^2+^/g]	ABTS [µM TE/g]
BC-S/L-1	73.51 ± 0.92 ^g^	59.66 ± 0.06 ^j^	146.34 ± 1.83 ^j^
BC-S/L-2	75.81 ± 0.92 ^f^	61.56 ± 0.33 ^i^	154.19 ± 1.44 ^h, i^
BC-S/L-3	59.82 ± 1.06 ^h^	57.17 ± 0.32 ^k^	101.99 ± 1.83 ^k^
BC-UAE-1	86.10 ± 0.90 ^d^	75.46 ± 0.21 ^c^	151.65 ± 2.23 ^i, j^
BC-UAE-2	80.74 ± 0.64 ^e^	69.39 ± 0.32 ^g, h^	157.43 ± 1.39 ^h^
BC-UAE-3	75.55 ± 0.88 ^f, g^	71.35 ± 0.36 ^f^	171.52 ± 2.23 ^g^
BC-UAE-4	76.74 ± 0.39 ^f^	70.04 ± 0.32 ^g^	174.75 ± 1.39 ^g^
BC-UAE-5	94.52 ± 0.59 ^b, c^	73.08 ± 0.31 ^e^	207.78 ± 1.74 ^c^
BC-MAE-1	96.39 ± 0.82 ^b^	87.12 ± 0.39 ^a^	222.79 ± 2.23 ^a^
BC-MAE-2	86.02 ± 0.51 ^d^	78.32 ± 0.26 ^b^	209.39 ± 1.39 ^b, c^
BC-MAE-3	75.30 ± 0.44 ^f, g^	57.24 ± 0.31 ^k^	155.81 ± 1.74 ^h, i^
BC-PLE-1	94.10 ± 1.03 ^c^	87.50 ± 0.12 ^a^	196.00 ± 2.62 ^e^
BC-PLE-2	92.82 ± 0.64 ^c^	72.63 ± 0.22 ^e^	201.54 ± 0.80 ^d^
BC-PLE-3	81.85 ± 0.53 ^e^	68.97 ± 0.26 ^h^	159.51 ± 1.39 ^h^
BC-PLE-4	92.57 ± 0.39 ^c^	74.56 ± 0.26 ^d^	185.84 ± 2.08 ^f^
BC-PLE-5	107.7 ± 0.92 ^a^	76.08 ± 0.27 ^c^	213.32 ± 0.80 ^b^

DPPH—2,2-diphenyl-1-picrylhydrazyl assay; FRAP—ferric reducing antioxidant power assay; ABTS—2,2′-azino-bis(3-ethylbenzthiazoline-6-sulfonic acid assay, TE—Trolox equivalent; Fe^2+^—ferrous ion equivalent; B—black currant; S/L—conventional solid/liquid extraction; UAE—ultrasound-assisted extraction; MAE—microwave-assisted extraction; PLE—pressurized liquid extraction. Results are expressed as mean ± standard deviation (SD). Tukey’s multiple comparison test was performed at *p* < 0.05, and different letters represent statistically significant differences among samples.

**Table 3:** Antioxidant activity obtained by DPPH, FRAP, and ABTS assays of red currant extracts obtained by conventional and sustainable extraction techniques.

Sample	DPPH [µM TE/g]	FRAP [µM Fe^2+^/g]	ABTS [µM TE/g]
RC-S/L-1	58.46 ± 0.68 ^i^	48.52 ± 0.39 ^f^	106.38 ± 2.62 ^f^
RC-S/L-2	56.76 ± 0.29 ^i^	43.03 ± 0.36 ^i^	90.22 ± 2.40 ^h^
RC-S/L-3	49.53 ± 0.26 ^k^	40.10 ± 0.43 ^j^	73.12 ± 2.43 ^j^
RC-UAE-1	70.88 ± 1.03 ^f^	52.07 ± 0.37 ^d^	103.38 ± 1.20 ^f, g^
RC-UAE-2	74.96 ± 0.78 ^e^	46.41 ± 0.49 ^g^	138.72 ± 3.02 ^c^
RC-UAE-3	63.39 ± 0.64 ^h^	49.90 ± 0.27 ^e^	80.98 ± 1.44 ^i^
RC-UAE-4	67.81 ± 0.39 ^g^	51.55 ± 0.36 ^d^	97.14 ± 2.08 ^g^
RC-UAE-5	85.42 ± 0.78 ^c^	56.59 ± 0.32 ^c^	187.91 ± 2.40 ^a^
RC-MAE-1	93.76 ± 1.06 ^b^	63.70 ± 0.30 ^a^	155.58 ± 1.44 ^b^
RC-MAE-2	96.56 ± 0.53 ^a^	57.24 ± 0.18 ^c^	126.94 ± 2.77 ^d^
RC-MAE-3	73.26 ± 0.92 ^e^	46.89 ± 0.18 ^g^	115.85 ± 2.08 ^e^
RC-PLE-1	78.62 ± 0.44 ^d^	64.56 ± 0.33 ^a^	142.18 ± 2.08 ^c^
RC-PLE-2	94.18 ± 0.77 ^b^	56.45 ± 0.42 ^c^	158.35 ± 1.06 ^b^
RC-PLE-3	70.62 ± 0.59 ^f^	44.48 ± 0.12 ^h^	114.47 ± 3.02 ^e^
RC-PLE-4	53.35 ± 0.68 ^j^	31.89 ± 0.10 ^k^	81.90 ± 2.08 ^i^
RC-PLE-5	84.48 ± 0.68 ^c^	59.45 ± 0.42 ^b^	159.51 ± 0.80 ^b^

DPPH—2,2-diphenyl-1-picrylhydrazyl assay; FRAP—ferric reducing antioxidant power assay; ABTS—2,2′-azino-bis(3-ethylbenzthiazoline-6-sulfonic acid assay, TE—Trolox equivalent; Fe^2+^—ferrous ion equivalent; RC—red currant; S/L—conventional solid/liquid extraction; UAE—ultrasound-assisted extraction; MAE—microwave-assisted extraction; PLE—pressurized-liquid extraction. Results are expressed as mean ± standard deviation (SD). Tukey’s multiple comparison test was performed at *p* < 0.05, and different letters represent statistically significant differences among samples.

## Data Availability

Data are contained within the article.

## Supplementary Materials

The following supporting information can be downloaded at: www.mdpi.com/article/10.3390/foods11030325/s1, Table S1: Total extraction yield (Y) of black and red currant extracts obtained by conventional and sustainable extraction techniques; Table S2: Total phenolic content (TPC) of black and red currant extracts obtained by conventional and sustainable extraction techniques; Table S3: Total flavonoid content (TF) of black and red currant extracts obtained by conventional and sustainable extraction techniques; Table S4: Total monomeric anthocyanin content (TMAC) of black and red currant extracts obtained by conventional and sustainable extraction techniques.
