# Supplementary material for: Sustainable Extractions for Maximizing Content of Antioxidant Phytochemicals from Black and Red Currants
Source: Foods. 2022 Jan 24;11(3):325. doi: 10.3390/foods11030325 (PMC8833918; doi:10.3390/foods11030325)
Supplement: Supplementary file 1 [file foods-11-00325-s001.zip › foods-1520610-supplementary.pdf]

Table S1. Total extraction yield (Y) of black and red currant extracts obtained by conventional and sustainable extraction techniques

Black currant			Red currant		
Sample	Y [%]	std	Sample	Y [%]	std
BC-S/L-1	54.34^{e,f,g,h,i}	0.54	RC-S/L-1^{d,e}	47.56	0.47
BC-S/L-2	53.44^{g,h,i}	0.61	RC-S/L-2^{c,d}	48.85	0.24
BC-S/L-3	53.97^{f,g,h,i}	0.87	RC-S/L-3^{e}	45.89	1.02
BC-UAE-1	56.21^{d,e,f,g}	0.87	RC-UAE-1^{b}	53.35	0.25
BC-UAE-2	57.18^{c,d,e,f}	0.36	RC-UAE-2^{b}	53.22	0.18
BC-UAE-3	68.43^{a}	0.73	RC-UAE-3^{c,d}	49.60	0.62
BC-UAE-4	52.21^{h,i}	0.29	RC-UAE-4^{d,e}	47.46	1.23
BC-UAE-5	57.70^{c,d,e}	0.95	RC-UAE-5^{c}	50.11	0.53
BC-MAE-1	61.68^{b}	0.25	RC-MAE-1^{c,d}	49.40	1.11
BC-MAE-2	51.38^{i}	3.24	RC-MAE-2^{c,d,e}	47.89	0.41
BC-MAE-3	47.19^{j}	0.22	RC-MAE-3^{e}	46.24	0.48
BC-PLE-1	47.54^{j}	1.04	RC-PLE-1^{c,d}	49.46	1.49
BC-PLE-2	60.10^{b,c}	1.35	RC-PLE-2^{a}	56.04	0.82
BC-PLE-3	55.62^{d,e,f,g,h}	0.50	RC-PLE-3^{a,b}	54.79	0.18
BC-PLE-4	58.18^{c,d}	1.67	RC-PLE-4^{f}	38.49	0.45
BC-PLE-5	58.75^{b,c,d}	0.33	RC-PLE-5^{b}	52.62	0.98

Results were expressed as mean ± standard deviation (SD). Tukey's multiple comparison test was performed at $p<0.05$ and different letters represent statistically significant differences among samples.

Table S2. Total phenolic content (TPC) of black and red currant extracts obtained by conventional and sustainable extraction techniques

Black currant			Red currant		
Sample	TP [g GAE/100 g]	std	Sample	TP [g GAE/100 g]	std
BC-S/L-1	2.0849^{h}	0.0243	RC-S/L-1	1.6646^{j}	0.0070
BC-S/L-2	2.2219^{g}	0.0039	RC-S/L-2	1.6399^{j}	0.0085
BC-S/L-3	1.9389^{i}	0.0217	RC-S/L-3	1.3232^{k}	0.0108
BC-UAE-1	2.6688^{d}	0.0103	RC-UAE-1	2.0587^{f}	0.0070
BC-UAE-2	2.7227^{d}	0.0170	RC-UAE-2	2.0621^{f}	0.0103
BC-UAE-3	2.9428^{c}	0.0170	RC-UAE-3	1.7544^{i}	0.0103
BC-UAE-4	2.5947^{e}	0.0318	RC-UAE-4	1.8892^{h}	0.0039
BC-UAE-5	2.9630^{c}	0.0103	RC-UAE-5	2.1284^{e}	0.0118

BC-MAE-1	3.4122^{a}	0.0067	RC-MAE-1	2.3777^{c}	0.0070
BC-MAE-2	2.9091^{c}	0.0140	RC-MAE-2	2.2979^{d}	0.0051
BC-MAE-3	2.3768^{f}	0.0237	RC-MAE-3	1.9700^{g}	0.0103
BC-PLE-1	2.9563^{c}	0.0217	RC-PLE-1	2.3889^{c}	0.0039
BC-PLE-2	3.0237^{b}	0.0206	RC-PLE-2	2.5978^{b}	0.0118
BC-PLE-3	2.6059^{e}	0.0170	RC-PLE-3	1.9038^{h}	0.0019
BC-PLE-4	3.0574^{b}	0.0039	RC-PLE-4	1.2738^{l}	0.0070
BC-PLE-5	2.9496^{c}	0.0237	RC-PLE-5	2.6303^{a}	0.0070

Results were expressed as mean ± standard deviation (SD). Tukey's multiple comparison test was performed at $p<0.05$ and different letters represent statistically significant differences among samples.

Table S3. Total flavonoid content (TF) of black and red currant extracts obtained by conventional and sustainable extraction techniques

Black currant			Red currant		
Sample	TF [g CE/100 g]	std	Sample	TF [g CE/100 g]	std
BC-S/L-1	0.4903^{f}	0.0025	RC-S/L-1	0.4003^{j}	0.0006
BC-S/L-2	0.4774^{f}	0.0025	RC-S/L-2	0.3913^{k}	0.0016
BC-S/L-3	0.4717^{f}	0.0033	RC-S/L-3	0.3476^{l}	0.0033
BC-UAE-1	0.6372^{e}	0.0054	RC-UAE-1	0.5221^{f}	0.0021
BC-UAE-2	0.6529^{e}	0.0037	RC-UAE-2	0.5027^{h,i}	0.0028
BC-UAE-3	0.8321^{a}	0.0050	RC-UAE-3	0.5289^{f}	0.0035
BC-UAE-4	0.6522^{e}	0.0066	RC-UAE-4	0.5432^{e}	0.0038
BC-UAE-5	0.7841^{b}	0.0099	RC-UAE-5	0.5106^{g,h}	0.0035
BC-MAE-1	0.7934^{b}	0.0081	RC-MAE-1	0.4992^{i}	0.0016
BC-MAE-2	0.6873^{d}	0.0078	RC-MAE-2	0.5636^{d}	0.0025
BC-MAE-3	0.7038^{d}	0.0045	RC-MAE-3	0.5511^{e}	0.0021
BC-PLE-1	0.7640^{c}	0.0087	RC-PLE-1	0.5124^{g}	0.0039

BC-PLE-2	0.6515[e]	0.0054	RC-PLE-2	0.5837[c]	0.0045
BC-PLE-3	0.7991[b]	0.0078	RC-PLE-3	0.6453[b]	0.0006
BC-PLE-4	0.6916[d]	0.0078	RC-PLE-4	0.3232[m]	0.0032
BC-PLE-5	0.7941[b]	0.0089	RC-PLE-5	0.7743[a]	0.0027

Results were expressed as mean ± standard deviation (SD). Tukey's multiple comparison test was performed at p<0.05 and different letters represent statistically significant differences among samples.

Table S4. Total monomeric anthocyanin content (TMAC) of black and red currant extracts obtained by conventional and sustainable extraction techniques

Black currant			Red currant		
Sample	TMAC [mg CGE/100 g]	std	Sample	TMAC [mg CGE/100 g]	std
BC-S/L-1	60.73[j]	0.54	RC-S/L-1	8.49[g,h,i]	0.05
BC-S/L-2	85.89[d]	2.22	RC-S/L-2	9.28[e,f]	0.06
BC-S/L-3	81.66[e]	1.17	RC-S/L-3	9.45[e,f]	0.17
BC-UAE-1	69.52[g,h]	0.19	RC-UAE-1	8.33[h,i,j]	0.05
BC-UAE-2	90.06[c]	1.39	RC-UAE-2	11.05[b]	0.13
BC-UAE-3	96.07[b]	1.83	RC-UAE-3	9.73[d,e]	0.35
BC-UAE-4	80.88[e]	0.35	RC-UAE-4	11.64[a]	0.22
BC-UAE-5	80.71[e]	0.26	RC-UAE-5	10.28[c,d]	0.32
BC-MAE-1	104.09[a]	1.01	RC-MAE-1	9.59[e]	0.17
BC-MAE-2	76.15[f]	1.59	RC-MAE-2	8.96[f,g]	0.07
BC-MAE-3	69.58[g,h]	0.82	RC-MAE-3	10.38[c]	0.14
BC-PLE-1	71.64[g]	1.93	RC-PLE-1	8.02[i,j]	0.17
BC-PLE-2	66.41[h,i]	0.26	RC-PLE-2	8.62[g,h]	0.12
BC-PLE-3	59.11[j]	1.17	RC-PLE-3	7.76[j,k]	0.10
BC-PLE-4	64.90[i]	1.26	RC-PLE-4	7.20[k]	0.35
BC-PLE-5	59.95[j]	1.20	RC-PLE-5	8.52[g,h,i]	0.12

Results were expressed as mean ± standard deviation (SD). Tukey's multiple comparison test was performed at p<0.05 and different letters represent statistically significant differences among samples.
